# The Management of Delirium

**Published:** 1875-03

**Authors:** 


THE MANAGEMEMT OF DELIRIRUM.—Dr. J. M. Fothergill (*Practitioner*, Dec., 1874,) after discussing the psychical treatment of delirium, makes the following points as to its physical treatment. That form that marks commencing fevers in children scarcely calls for treatment, being a mere symptomatic affair. In delirium tremens, if the pulse be full, bounding and incompressible, a good dose of opium with antimony is indicated. But if the pulse be small, compressible and very quick, when sleep is kept off by that irritability which is associated with commencing exhaustion in nerve structures, full doses of opium are dangerous. Perhaps death here results from the effect of the opium upon the cardiac ganglia. In such cases half-ounce doses of tincture of digitalis have been given with good effect. Bromide of potassium with a vegetable narcotic might be combined with the digitalis.

In sustained pyretic conditions, if there be considerable vascular excitement and heat of head, opiates are inadmissible.

Chloral in fifteen-grain hourly doses until a drachm is taken, acting as it does upon the vascular system as well as upon the nerve centres, is better. Subcutaneous injections, of a strength not exceeding ten grains to the ounce, will often be useful in typhoid, non-absorbing conditions.

High temperature usually affects intelligence, and the local and general application of cold is of service. There is a form of delirium, which is more properly cerebral exhaustion, met with after the acute pyretic stage is over. Where there is persistent watchfulness without much vascular excitement, where delirium remains after a cerebral inflammation has been subdued, or where the sensorium is excited without heat of scalp or much throbbing of the arteries of the head, opium or chloral, or opium and chloral, are indicated.

